# Preparation, Characterization, and Antioxidant Activity Evaluation of Liposomes Containing Water-Soluble Hydroxytyrosol from Olive

**DOI:** 10.3390/molecules22060870

**Published:** 2017-05-24

**Authors:** Jiao-Jiao Yuan, Frank G.F. Qin, Jun-Ling Tu, Bing Li

**Affiliations:** 1 School of Chemical Engineering and Energy Technology, Dongguan University of Technology, Dongguan 523808, China; yuanjj@dgut.edu.cn; 2 School of Food Science and Engineering, South China University of Technology, Guangzhou 510640, China; lcbingli@scut.edu.cn

**Keywords:** hydroxytyrosol, liposomes, preparation, slow release, antioxidant activity

## Abstract

Due to the multiple hydroxyl groups in its structure, hydroxytyrosol (HT) is very sensitive to air and light and has very strong instability and hydrophilicity that affect its biological activity. This study attempted to prepare liposomes containing water-soluble HT to improve the bioavailability and biocompatibility of the target drug. The preparation process factors (temperature, mass ratio of phospholipid (PL) and cholesterol (CH), Tween-80 volume, HT mass) were studied and response surface methodology (RSM) was applied to optimize the conditions. The results demonstrated that by using a temperature of 63 °C, mass ratio of PL and CH 4.5:1, HT mass 5 mg and Tween-80 volume of 6 mL, HT liposomes with an encapsulation efficiency (EE) of 45.08% were prepared. It was found that the particle sizes of the HT liposomes were well distributed in the range of 100–400 nm. Compared to free HT, prepared HT liposomes had better stability and a distinct slow release effect in vitro. Besides, HT liposomes presented better DPPH radical scavenging activity than free HT, which could be due to the fact that HT was encapsulated fully inside the liposomes. In addition, the encapsulation mechanism of HT was evaluated. In summary, the results indicated that HT liposome could enhance the antioxidant activity and was a promising formulation for prolonging the biological activity time of the target drug.

## 1. Introduction

Hydroxytyrosol (HT, 3,4-dihydroxyphenethyl alcohol), is present in olive oil and olive leaves [1,2] as an important active compound. With three free hydroxyl groups, HT has antioxidant [3,4], anti-inflammatory [5], cardioprotective [6,7], and anti-microbial properties [8,9], and in particular, anti-tumor activity to inhibit the proliferation of human promyelocytic leukemia HL60 cells [10], human colon cancer HT-29 cells [11] and MCF-7 human breast cancer cells [12]. Therefore, HT could be used as a potentially safe natural food additive, cosmetic ingredient and medicine. Research on the chemical structure of HT and its derivatives [13,14], biological activity [15], pharmacokinetics and mechanism of action [16] as an anti-cancer drug or anti-cancer adjuvant have been reported. Due to the multiple hydroxyl groups in its structure, HT is very sensitive to air and light and has very strong instability and hydrophilicity that affect its biological activity, causing its easy release and rapid elimination from the body. Owing to these physical-chemical properties and low bioavailability, it is difficult to mix into foods or drug formulations and the quality of relevant preparations is also greatly affected.

One of the strategies to overcome the rapid release of HT and increasing its pharmacological bioactivity in vivo is to encapsulate it in a suitable delivery system [17]. Recently, water in oil (W/O) emulsions, microemulsions [18] and nanoparticles [19] were shown to be effective carriers of HT, as a natural antioxidant, which retained its scavenging ability inhibiting approximately 90% of the free radical within the first 5 min of incubation. In addition, liposomes, a directed drug carrier for targeted drug-delivery systems, is a new drug formulation generally capable of encapsulating lipid-soluble substances [20,21,22,23]. A few references have reported that water-soluble target-drugs could be encapsulated in liposomes, improving their lipid solubility [24]. However, the preparation and property evaluation of HT liposomes has not been investigated so far.

Liposomes are composed of a lipid bilayer with closed vesicles [25] and were first used as a new drug formulation form in 1971. As drug delivery carriers, liposomes have many fine properties that improve the drug availability, such as targeting, slow release, and protective features. Firstly, prepared liposomes generally locate to the kidney, liver, spleen, and other mononuclear rich organs, and liposomes could be engulfed by mononuclear phagocytes [26]. The folate-targeting liposomes of encapsulated anti-sarcosine antibodies presented competitive inhibitory effects [27]. Secondly, release of drugs from liposomes could be slower than that of the free drug, and action time of the drugs was also thus prolonged [28]. Compared to the free drug, liposome encapsulated drug accumulation in the heart and kidney was significantly lower [29]. Lastly, the coated drug is protected and its stability may be increased when encapsulated inside liposomes [30]. It was reported that doxorubicin encapsulated inside liposomes could be protected and showed cardiotoxicity decrease while maintaining anticancer potency [31]. Phospholipid (PL) and cholesterol (CH), with good biocompatibility and biodegradability, are used as liposome wall materials, and are able to enhance the bioactivity by improving drug solubility and bioavailability, in vitro and in vivo stability, as well as preventing interference with the external environment. It is therefore that liposomes have potential applications in a vast range of fields, including medicine (e.g., diagnosis, cancer therapy, gene delivery), cosmetics, food technology and agriculture. In this study, HT was encapsulated with liposomes by a film dispersion method. Since there are various parameters affecting liposome formation and emulsification such as temperature, mass ratio of PL and CH, Tween-80 volume, HT mass, etc., it is necessary to select the most important factors and to gain full knowledge of the interactions among these factors and, finally, to optimize this process by the RSM method. Based on the optimal conditions, the stability, slow release characterization and antioxidant effect of the HT liposomes were investigated. The aim of the study was thus to evaluate the properties of HT liposome and improve the bioavailability, biocompatibility and stability of the HT drug.

## 2. Results and Discussion

### 2.1. Preparation of HT Liposomes

#### 2.1.1. Effect of Temperature

**Figure 1:**
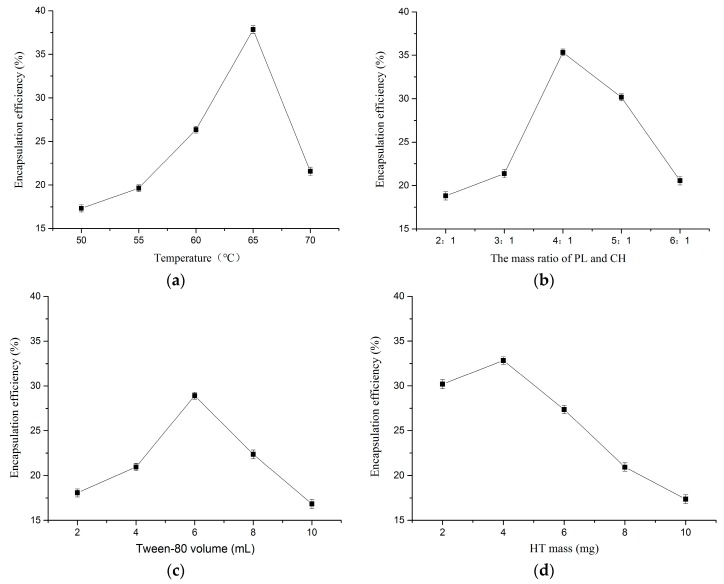
Effect of factor on EE (**a**) temperature; (**b**) mass ratio of PL and CH; (**c**) tween-80 volume; (**d**) HT mass (*n* = 3).

The effect of temperature on HT liposome preparation is shown in Figure 1a. Preparation experiments were carried out at different temperatures (50, 55, 60, 65, 70 °C), while the other parameters were fixed as follows: mass ratio of PL and CH 4:1, Tween-80 volume 6 mL, HT mass 4 mg. From the relationship between temperature and EE, the tendency increased in a steep manner when the temperature ranged from 50 to 65, then decreased straightly as the temperature increased further. When the temperature was 65 °C, EE was the highest at 37.83%. This might be due to a temperature effect on lipid bilayer film formation. When the temperature was low, the speed of the lipid bilayer film formation could be slow, then the lipid material spent a long time in organic solvent and the formed film density was poor. However, when the temperature was high, the speed of the lipid bilayer film formation could be fast, and lipid materials did not have enough time to mix with each other fully, then HT aqueous solution was added to produce rapid hydration, which resulted in a loose bilayer structure. It was thus demonstrated that the HT liposomes preparation process was not fit for higher temperature.

#### 2.1.2. Effect of Mass Ratio of PL and CH

Figure 1b presents the effect of mass ratio of PL and CH on the HT liposomes preparation. Preparation experiments were carried out at different mass ratios of PL and CH (2:1, 3:1, 4:1, 5:1, 6:1), while the other parameters were as follows: temperature 60 °C, Tween-80 volume 6 mL, HT mass 4 mg. It was found that the EE was sharply increased with increasing mass ratio, but with the further increase of mass ratio, EE was shown to decline sharply, and the optimum mass ratio for EE was 4:1 (as seen in Figure 1b). CH is the main component of liposome bilayer membrane material, and it is a good membrane fluidity regulator, able to improve EE and liposome stability. This might be explained by the fact that if the ratio was small, CH content was relatively high, and the difficulty of membrane formation would be increased, and the formed film is relatively flexible. If the ratio was big, the membrane fluidity of the liposomes would be affected.

#### 2.1.3. Effect of Tween-80 Volume

Figure 1c shows the effect of Tween-80 volume on HT liposome preparation. Preparation experiments were carried out at different Tween-80 volumes (2, 4, 6, 8, 10 mL), while the other parameters were fixed as follows: temperature 60 °C, mass ratio of PL and CH 4:1, HT mass 4 mg. As shown in Figure 1c, the EE increased to reach its maximum value (28.91%) at 6 mL. Tween-80 is a kind of nonionic hydrophilic surfactant, which contains both hydrophilic and hydrophobic groups. Liposomes and Tween-80 mixed together, and the surface of the liposome could build a “stereo” barrier layer by physical adsorption, so the liposomes containing Tween-80 had better stability, but a high content of Tween-80 might lead to coated HT leakage.

#### 2.1.4. Effect of HT Mass

HT liposome preparations were carried out using different HT masses (2, 4, 6, 8, 10 mg) while the other parameters were held as follows: temperature 60 °C, mass ratio of PL and CH 4:1, Tween-80 volume 6 mL. As shown in Figure 1d, the highest EE (32.84%) was found for 4 mg HT. When the HT quality was high, the relative proportion of lipid membranes and HT would be out of balance, and the HT could not be completely encapsulated.

#### 2.1.5. Optimization of HT Liposomes Preparation by RSM

Based on the results of single factor experiments, the effect of Tween-80 volume on the HT content was not significant. Therefore, a 17-run BBD was used to optimize the three independent variables, including *X*_1_ (temperature), *X*_2_ (mass ratio of PL and CH) and *X*_3_ (HT mass). In order to study the combined effect of these factors, investigations were performed to use different combinations of the physical parameters. Table 1 shows the experimental design and dependent variable (*Y*, EE).

The EE obtained at various levels of the three independent variables (*X*_1_, *X*_2_, and *X*_3_) were subjected to multiple regression to obtain a second-order polynomial Equation (1), as follows:

*Y* = 44.13 − 0.49*X*_1_ + 0.41*X*_2_ + 0.29*X*_3_ − 0.58*X*_1_*X*_2_ − 1.06*X*_1_*X*_3_ − 0.22*X*_2_*X*_3_ − 1.58*X*_1_^2^ − 0.49*X*_2_^2^ − 0.59*X*_3_^2^ (1)

where *Y* represents EE (%); *X*_1_, *X*_2_ and *X*_3_ represent temperature, mass ratio of PL and CH, and HT mass, respectively.

According to the regression model equation, the fitting coefficient of three variables showed values of 0.49 > 0.41 > 0.29, implying that the *X*_1_ (temperature) and *X*_2_ (mass ratio of PL and CH) were the main variables in the HT liposomes preparation process.

The statistical parameters obtained from the variance analysis of optimization model are given in Table 2. The model *F*-value of 41.57 and *p* < 0.001 implied the model was significant, and the model could predict the real experimental data. Meanwhile, the value of the determination coefficient (*R*^2^ = 0.9816) and the adjusted determination coefficient (*R*_adj_^2^ = 0.9580) also confirmed that the model was statistically significant. Additionally, the lack of fit was not significant (*p* > 0.05), which implied that the calculated values could be fit with the experimental values. Adep precision measured the signal to noise ratio and the ratio greater than 4 was desirable. Thus, ratio of 17.696 indicated an adequate signal. In conclusion, the model was found to be adequate for navigating the design space.

The *p*-value is used as a tool to check the significance of each coefficient, which in turn indicates the interaction strength between each independent variable. It could be observed that the coefficients of *X*_1_, *X*_1_*X*_3_, *X*_1_^2^, were highly significant (*p* < 0.001). Meanwhile, the coefficients of *X*_2_, *X*_1_*X*_2_, *X*_2_^2^ and *X*_3_^2^ were found to be significant (*p* < 0.01), and *X*_3_ was found to be significant (*p* < 0.05). Moreover, the coefficient of *X*_2_*X*_3_ was found to be non-significant (*p* > 0.05). The results were demonstrated that *X*_1_ and *X*_2_ variables had a significant effect on liposome preparation. For the *X*_1_*X*_2_ interaction, *p* < 0.05, implied that the temperature was related to mass ratio of PL and CH in the preparation process. Moreover, for the *X*_1_*X*_3_ interaction, *p* < 0.001, implied that the temperature was closely related to HT mass in the preparation process.

**Figure 2:**
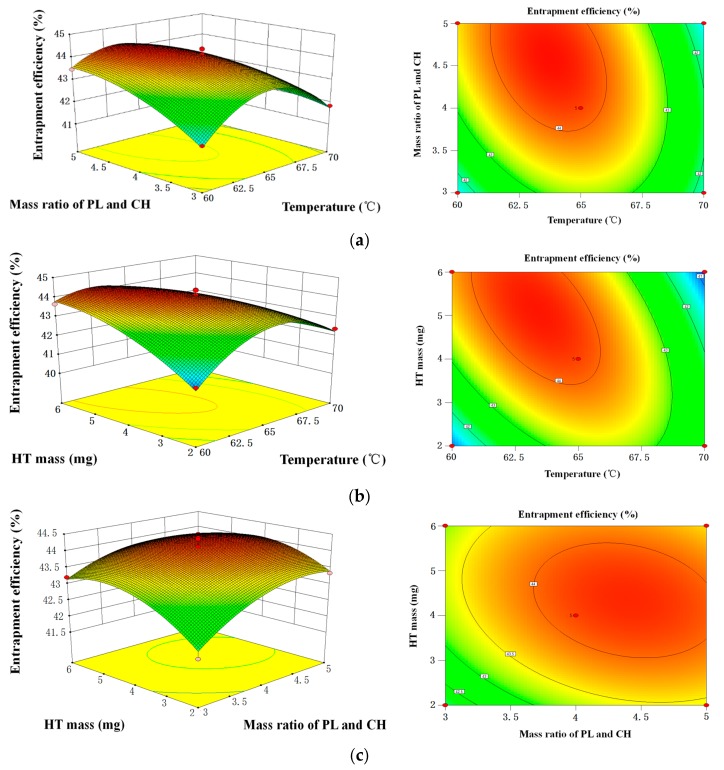
Response surface plots and contour plots of the interactive effects on the encapsulation efficiency of HT liposomes (**a**) mass ratio of PL and CH and temperature of the interactive effects; (**b**) HT mass and temperature of the interactive effects; (**c**) HT mass and mass ratio of PL and CH of the interactive effects.

As shown in Figure 2, the contour plots and 3D response surface plots, obtained by the Design Expert software, are the graphical representations of the regression. In the contour plots and 3D response surface plots, the EE of HT liposome was obtained along with two continuous variables, while the other variables were fixed constant at their 0 levels. In the figures, the maximum predicted value indicated by the surface was confined in the smallest ellipse in the contour diagram. Elliptical contours were obtained when there was a perfect interaction between the independent variables.

The EE affected by the temperature and mass ratio of PL and CH was seen in Figure 2a, when HT mass was maintained at 0 level. It was obvious that the EE increased as the temperature was increased from 60 to 70 °C. Subsequently, the EE gradually decreased. The EE also increased as mass ratio of PL and CH increased from 3:1, then the EE slightly reduced after ratio of 4:1. Figure 2b shows the EE varied with temperature and HT mass. The maximum EE was obtained when temperature and HT mass were 65 °C and 4 mg, respectively. The EE affected by mass ratio of PL and CH and HT mass was shown in Figure 2c, whereas the variable (temperature) was fixed at 0 level. The round contour indicated that the interaction effect between these factors was not significant. It was agreement with the *p*-value of *X*_1_*X*_3_. The maximum EE was obtained when mass ratio of PL and CH and HT mass were 4:1 and 4 mg, respectively.

After the 17-run BBD of preparation parameters, the optimal conditions for achieving maximal yield of EE obtained by the regression were as follows: temperature 62.83 °C, mass ratio of PL and CH 4.56:1, HT mass 5.05 mg and Tween-80 volume 6 mL. The predicted yield value was 44.43%. Considering the practical experiment and production, the optimal parameters were adjusted to temperature 63 °C, mass ratio of PL and CH 4.5:1, HT mass 5 mg and Tween-80 volume 6 mL. Under these conditions, the experimental yield of HT was (45.08 ± 0.56) % (*n* = 3).

### 2.2. Mean Particle Size and Stability

**Figure 3:**
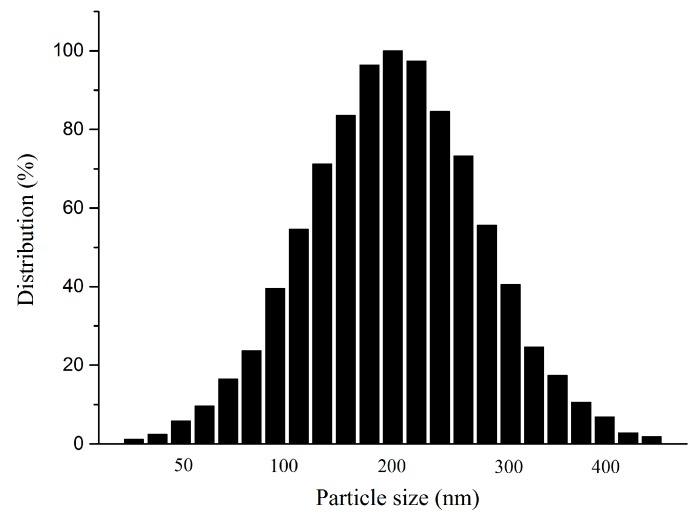
The size distribution of HT liposome.

The particle size of liposomes is an important property, as it could affect liposome stability and bioavailability efficiency. Therefore, the liposomes prepared using the above optimal conditions were expected to enhance the stability and residence time of drugs in the circulation, and be well-distributed to tissues. From Figure 3 we could see that the particle sizes of HT liposomes were distributed in the range of 100–400 nm, and the average particle size was 200 nm. Considering the results, it was clear that liposomes had a uniform distribution, presenting a homogeneous milk-white and translucent suspension.

The centrifugation results showed that HT liposomes were not destroyed by high speed centrifugation and no visible change to the physical appearance in the treatment process was noted. In addition, the liposomal formulations showed excellent physical stability and drug retention during this period.

**Figure 4:**
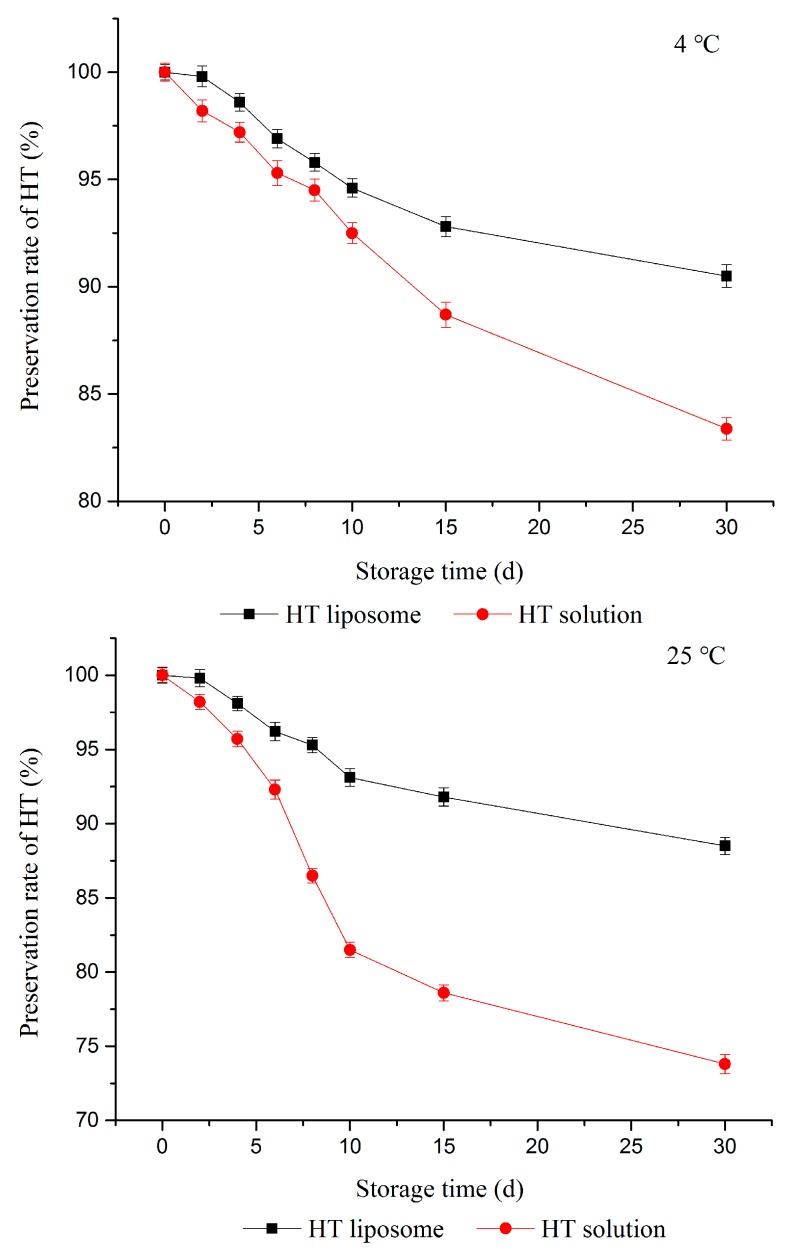
Stability of HT liposome and HT solution at 4 °C and 25 °C.

As shown in Figure 4, the preservation rate of HT in pH 7.4 PBS solution were insignificantly (*p* > 0.05) changed at 4 °C, but the preservation rate had a tendency to be slightly decreased as time went by. The preservation rate of HT in solution was 83.38% at 4 °C. At the same time, the preservation rate of HT in HT solution decreased to 73.8% at 25 °C.

The preservation rate of HT in HT solution was both distinctly less than HT liposomes at 4 °C and 25 °C over 30 days (*p <* 0.05). The results showed that the prepared liposomes in pH 7.4 PBS solution were stable at least for 30 d at 4 °C and 25 °C, but the corresponding free HT was stable for 12 days at 4 °C and 7 days at 25 °C (standard for 90% preservation rate). It should be noted that storage temperature and formulations both had influence on the storage stability of HT. It was considered that liposomes could further improve HT stability, whereby HT was attached to the surface and inside.

### 2.3. In Vitro Slow Release Study

**Figure 5:**
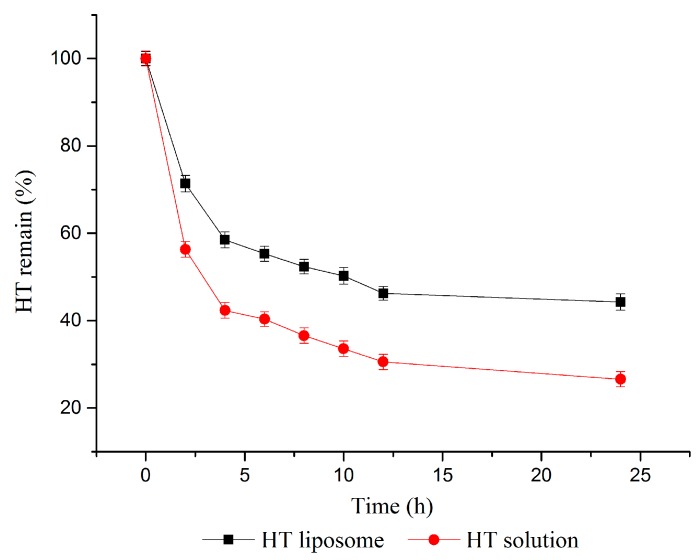
Slow release of HT liposome and HT solution in vitro.

The in vitro slow release of HT liposomes was examined by imitating the human body environment. Figure 5 presents the HT release profiles from HT liposomes and HT solution in pH 7.4 phosphate buffer medium in vitro, and the release tendency of HT solution and HT liposomes was similar. In the period, the amount of HT released in solution was more than that of HT liposomes, and the remaining HT was 56.34 ± 1.82% and 71.38 ± 1.87% at 2 h, 26.59 ± 1.72% and 44.28 ± 1.88% at 24 h, respectively. However, the release ratio of HT in solution and liposome were both slow after 4 h. In brief, the release of HT in liposomes demonstrated significantly slower profiles compared to that of HT solution (*p* < 0.05). This result was likely due to the presence of lipid film which was located in HT material surface, and there was a distinct prolongation of HT release from HT-liposome. Therefore, the HT liposomes have potential application for controlled release into the human blood system. We expected that the prepared liposomes could increase the residence time of drug in the circulation.

### 2.4. DPPH Radical Scavenging Activity of HT Liposomes

The DPPH radical scavenging activity of HT liposomes and HT solution were determined. This kind of a comparison was deliberately performed to examine the effect of liposomes on the bioavailability of HT. Standard analysis of variance (ANOVA) was used for statistical evaluation of the data. Tukey tests were used to analyze the significance between HT solution and HT liposomes. All statistical tests were performed at the *p* < 0.05 level of significance.

As expected (in Table 3) the DPPH radical scavenging activity of HT solution and HT liposomes all presented a linear relationship among the different concentrations, and it was demonstrated that the antioxidant activity of the tested sample concentration exhibited distinct differences. Moreover, the different lowercase letters in a column meant that a significant difference was also discovered by ANOVA analysis (*p* < 0.05). However, the DPPH radical scavenging activity of HT solution and HT liposomes presented similar results at the same concentration, and the same capital letters in a row meant no significant difference was discovered by ANOVA analysis (*p* > 0.05). In other words, HT liposomes still retained the DPPH radical scavenging activity of HT, and it seemed that HT was encapsulated fully inside in the liposomes.

### 2.5. Encapsulation Mechanism of HT Liposomes

Chatzidaki [18] reported that W/O microemulsions and emulsions, as the carrier of HT, were successfully formulated with the addition of emulsifiers based on medium chain triglycerides. In the further research, findings were presented in [32] that indicated that the amounts of surfactants and emulsifiers both affected gastrointestinal lipolysis efficiency. In addition, the activity of pancreatic lipase displayed lower activity in the W/O microemulsions than that in the W/O emulsions. This was explained by the presence of higher amounts of emulsifiers (4.9% *w*/*w* lecithin and monoglycerides) in the composition of W/O microemulsions compared to W/O emulsions (1.3% *w*/*w* emulsifiers). Hence, the idea arose that preparation of liposomes, as a new carrier of HT, could be attempted in this study.

Lipophilicity is a key factor in many biological effects of compounds. The lipophilicity of HT was measured by determination of the octanol-water partition coefficient (*P*_o/w_ value), which was found to be 1.1 [33]. This meant that the partition concentration of HT was expected to be very similar in the water and the lipid phase, and therefore HT has amphiphilic character. Hence, it was supposed that HT could be encapsulated in the bilayers and interior of liposomes.

Guan [19] suggested that release of syringopicroside and HT from nanoparticles co-loaded with them fitted a sustained-release Higuchi equation, the mechanism of which might be explained by the fact that the nanoparticle carrier (monomethoxypolyethylene glycol-polylactide-glycolic acid) was a core–shell structure, and the drugs, incorporated into the nanoparticles, were released in a sustained manner via diffusion or degradation. As reported, liposomes were composed of a lipid bilayer with closed vesicles, similar to the core-shell structure, therefore, we supposed the encapsulation process of HT was also analogous.

**Figure 6:**
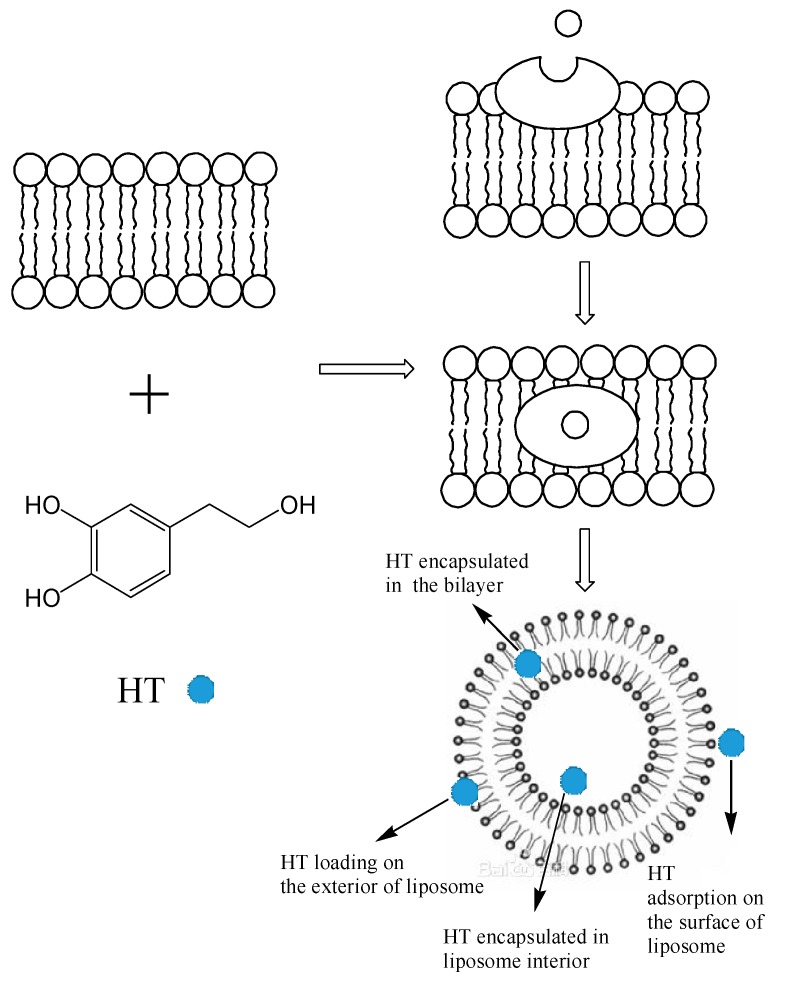
Encapsulation mechanism of liposomes with HT.

Based on above studies, the encapsulation mechanism of liposomes with HT was supposed to occur as indicated in Figure 6. When HT was mixed with blank liposomes, HT was adsorbed on the surface of the liposomes, then gradually loaded on the liposome exterior. As time went on, HT was encapsulated in the bilayer and interior of liposomes.

## 3. Materials and Methods

### 3.1. Material

HT standard (purity >98%, molecular weight 154) was purchased from Sigma (St. Louis, MO, USA). PL (from soybean, >98%) and CH were purchased from Aladdin Chemicals (Shanghai, China). Disodium hydrogen phosphate, sodium dihydrogen phosphate, Tween-80, ethyl alcohol and ethyl acetate were analytical reagent (Guangzhou Chemical Reagent Company, Guangzhou, China).

### 3.2. Methods

#### 3.2.1. Preparation of HT Liposomes

The HT liposomes were prepared using the film dispersion method [34]. PL, CH, Tween-80 and anhydrous ethanol were added to a round bottom flask with a certain ratio (mass), and dissolved with ultrasound to get a stable solution at a constant speed. Then, the ethanol solvent was removed by a vacuum rotary evaporator. At this point a layer of film was formed on the flask wall. In other words, a blank liposomes film was achieved. Briefly, a certain concentration of HT solution was mixed with blank liposome at a certain temperature. The liposome film dispersion was gently shaken and sonicated for 10 min. Finally, the HT liposome was filtered using 0.45 μm and 0.22 μm membranes successively.

#### 3.2.2. High Performance Liquid Chromatography (HPLC) Analysis

The identification and quantification analysis of HT was performed by HPLC [35]. The equipment was a SPD-20A instrument (Shimadzu, Kyoto, Japan) equipped with a DAD detector (280 nm). The column was a 5 μm BDS HYPERSIL C18 (250 × 4.6 mm, Thermo, Waltham, MA, USA), and its temperature was maintained at 30 °C. The mobile phase consisted of 0.2% phosphoric acid in water (A) and methanol (B) for a total running time of 20 min. The flow rate was 0.8 mL/min, and the injection volume was 10 μL. Identification analysis of HT was based on their HPLC spectrum and the retention time in comparison with standards analyzed under the same conditions. Furthermore, the quantification analysis of HT was based on the external calibration curves. The HT content had a good linear relationship with peak area in the range of 0.5586–8.9376 μg, and the regression equation was *y* = 1199063.90*x* + 119947.13 (*R*^2^ = 0.9996, *y* stands for peak area and *x* stands for HT content).

#### 3.2.3. The Determination of Encapsulation Efficiency

The EE of liposomes is an important characteristic to assess their quality. The EE of HT liposomes was measured in this study by the dialysis method with some modifications [36]. In brief, 50 mg HT liposome was added into a dialysis bag with a hydrophilic cellulose membrane (3000 molecular weight cut off, MWCO) to remove free HT. After 9 h of dialysis, the HT content was determined outside of the dialysis bag using the above HPLC method. Equation (2) was used to calculate the EE of HT liposomes as follows:

(2) $$\mathrm{EE}=\frac{C\times V-C_{0}\times V_{0}}{C\times V}\times 100\%$$

where EE is the encapsulation efficiency of the liposomes; 𝐶_0_ is the concentration of free HT in the dialysis filtrate; *V*_0_ is the volume of dialysis filtrate; 𝐶 is the total concentration of the HT solution; *V* is the volume of HT solution.

#### 3.2.4. Single Factor Experimental Design

The effects of temperature (50, 55, 60, 65, 70 °C), mass ratio of PL and CH (2:1, 3:1, 4:1, 5:1, 6:1), Tween-80 volume (2, 4, 6, 8, 10 mL), HT mass (2, 4, 6, 8, 10 mg) on EE of HT liposome were studied by a single factor design as follows: one factor was changed while the other factors were kept constant in each experiment. Every operation condition was repeated three times. The volume of prepared HT liposome was 10 mL, and the concentration of Tween-80 volume was 2 mg/mL.

#### 3.2.5. Box-Behnken Design

The Box-Behnken design (BBD) is an analytical method for optimization processes. It presents the relationship between factors and involved responses during the optimization of analytical systems. In this work, BBD was used to predict the levels of factors temperature (*X*_1_), mass ratio of PL and CH (*X*_2_), and HT mass (*X*_3_). Table 4 showed the input parameters and experimental design levels used. The experimental design consisted of a set of points lying at the midpoint of each edge and the replicated center points of the multidimensional cube.

#### 3.2.6. Determination of Mean Particle Size and Size Distribution

The dynamic light scattering (DLS) method was used to measure the mean particle size on a ZetaPlus laser particle analyzer and size distribution analysis of the prepared HT liposomes at 25 °C with a 90° scattering angle for optimum detection. One mL of liposome dispersion was dissolved in 5 mL of PBS (pH 7.4) using 0.45 μm membrane and the size of liposome was measured.

#### 3.2.7. Stability Tests

(1) Physical stability: HT liposomes were centrifuged 30 min by high speed centrifugation and any phenomena observed was recorded.
(2) Storage stability: EE of prepared liposomes in pH 7.4 PBS solution was measured over a 30-day period at 4 °C and 25 °C storage conditions. Samples were taken at different time intervals (0, 2, 4, 6, 8, 10, 15, 30 days) and EE were calculated immediately. Each experiment was repeated at least three times. The Equation (3) to calculate the preservation rate of HT was shown below:
  (3) $$w=\frac{EE_{i}}{EE_{0}}\times 100\%$$
  where *w* is the preservation rate of HT; *EE_i_* is the EE of HT liposome at time *i*; *EE*_0_ is the EE of HT liposomes prepared at the beginning.

#### 3.2.8. In Vitro Slow Release Effect Study

For the in vitro release test of prepared HT liposomes, remaining HT was measured by the dialysis method in saline medium [37]. HT liposome was diluted with normal saline medium and the final concentration of HT was adjusted to 2 mg/mL. A 2 mL sample was put into a dialysis tube (MWCO: 3000) which was placed into a 50 mL tube with 10 mL of dissolution medium. The tubes were incubated in a shaking water bath thermostat at 37 °C and shaken at a rate of 100 rpm by magnetic stirrer. At predetermined time intervals (2, 4, 6, 8, 10, 12, 24 h after starting incubation), whole medium (10 mL) was taken and the same volume of fresh medium (10 mL) was replaced. The amount of released HT was measured by HPLC. The release experiments were performed in triplicate. Equation (4) to calculate the HT remain was shown below:

(4) $$m=\frac{C_{1}\times V_{1}-C_{2}\times V_{2}}{C_{1}\times V_{1}}\times 100\%$$

where *m* is the HT remain; 𝐶_2_ is the concentration of free HT in the dialysis filtrate; *V*_2_ is the volume of dialysis filtrate; 𝐶_1_ is the total concentration of HT liposomes; *V*_1_ is the volume of HT liposomes.

#### 3.2.9. DPPH (1,1-diphenyl-2-picrylhydrazyl) Radical Scavenging Assay

The DPPH radical-scavenging effect was evaluated according to Yuan et al. [35]. 1 mL of each diluted sample was added to a 3 mL of ethanolic DPPH solution (40 μg/mL). After the two solutions were softly mixed and left for 30 min at room temperature, the optical density was measured at 517 nm by a spectrophotometer. The antioxidant activity of each sample was expressed in terms of DPPH scavenging capacity and calculated from the log-dose inhibition curve. Equation (5) was used to calculate the DPPH scavenging capacity as shown below:

(5) $$\eta =[1-\frac{A_{i}-A_{0}}{A_{1}}]\times 100\%$$

where *η* is the DPPH scavenging capacity of the sample; *A*_0_ is the absorbance with 1 mL sample and 3 mL ethanol after 30 min; *A*_1_ is the absorbance with 1 mL ethanol and 3 mL DPPH ethanol solution after 30 min (total radical); *A*_i_ is the absorbance with 1 mL sample and 3 mL DPPH ethanol solution after 30 min.

#### 3.2.10. Statistical Evaluation

The Design-Expert (version 7.1.3) statistical software (Stat-Ease, Minneapolis, MN, USA) was adopted to design experiment and analyze data. All calculated values were expressed as their mean ± S.D. and the figures were drawn by Origin 9.0 (OriginLab, Hampton, MA, USA). All data were analyzed for statistical significance by Student’s *t*-test with *p* < 0.05 indicating a significant difference by SPSS 11.0 (SPSS, Chicago, IL, USA).

## 4. Conclusions

Water-soluble HT encapsulated in liposomes was prepared by the film dispersion method, and the optimal conditions for liposome formation were obtained by RSM as follows: temperature 63 °C, mass ratio of PL and CH 4.5:1, HT mass 5 mg and Tween-80 volume 6 mL. The RSM result was reliable and the EE of HT was 45.08%. Besides, the particle sizes of HT liposomes were distributed in the range of 100–400 nm, and the average particle size was 200 nm. It was clear that liposomes were well-distributed. In addition, the stability properties showed the prepared HT liposomes had better stability than free HT solution. Compared to free HT, HT liposomes presented a distinctly slower release effect in vitro. In addition, HT liposomes still showed better DPPH radical scavenging activity than free HT, and it seemed that HT was fully encapsulated inside the liposomes. This indicated that HT liposomes could maintain or enhance the antioxidant activity of the drug and had the potential to act as delivery carrier for further improving the biological functions in vivo. This study provided new insights into methods and techniques for water-soluble active substances.

## Figures and Tables

**Table 1:** Box-Behnken experimental design and corresponding HT liposomes ^a^.

No.	*X*_1_ Temperature/°C	*X*_2_ Mass Ratio of PL and CH	*X*_3_ HT Mass/mg	*Y* EE/%
1	−1	0	1	43.65
2	0	−1	−1	41.91
3	−1	1	0	43.47
4	1	−1	0	41.83
5	1	0	−1	42.38
6	0	0	0	43.86
7	−1	−1	0	41.65
8	0	0	0	43.92
9	0	1	−1	43.34
10	0	0	0	44.35
11	1	0	1	40.56
12	0	0	0	44.39
13	-1	0	−1	41.25
14	0	0	0	44.13
15	1	1	0	41.32
16	0	1	1	43.75
17	0	−1	1	43.21

^a^ PH and CL: phospholipid and cholesterol.

**Table 2:** Variance analysis of items in regression equation.

Sources of Variation	Sum of Squares	*df*	Mean Square	*F* Value	*p* Value	Significance
model	23.90	9	2.66	41.57	<0.0001	***
*X*_1_	1.93	1	1.93	30.23	0.0009	***
*X*_2_	1.34	1	1.34	21.06	0.0025	**
*X*_3_	0.66	1	0.66	10.26	0.0150	*
*X*_1_*X*_2_	1.36	1	1.36	21.25	0.0025	**
*X*_1_*X*_3_	4.45	1	4.45	69.71	<0.0001	***
*X*_2_*X*_3_	0.20	1	0.20	3.10	0.1217	
*X*_1_^2^	10.48	1	10.48	164.06	<0.0001	***
*X*_2_^2^	0.99	1	0.99	15.51	0.0056	**
*X*_3_^2^	1.48	1	1.48	23.14	0.0019	**
residual	0.45	7	0.064			
lack of fit	0.21	3	0.071	1.23	0.4096	no
pure error	0.23	4	0.058			
total	24.34	16				
*R*^2^	0.9816					
*R*_adj_^2^	0.9580					
Adeq precision	17.696					

* *p* < 0.05; ** *p* < 0.01; *** *p* < 0.001.

**Table 3:** DPPH free radical scavenging activity of HT liposome and HT solution (*x* ± SD, *n* = 3).

Concentration (μg/mL)	DPPH Scavenging Capacity of HT Solution (%)	DPPH Scavenging Capacity of HT Liposome (%)
0.25	0.17 ± 0.08 eA	0.19 ± 0.06 eA
0.5	0.25 ± 0.13 dA	0.31 ± 0.93 dA
1	0.47 ± 0.16 cA	0.51 ± 0.43 cA
1.5	0.65 ± 0.12 bA	0.68 ± 0.29 bA
2	0.88 ± 0.18 aA	0.91 ± 0.61 aA

The same lowercase letter in a column and capital letter in a row showing a no difference (Tukey test, *p* > 0.05), and the different letter showing a significant difference (Tukey test, *p* < 0.05).

**Table 4:** Independent variables and their levels for Box-Behnken design.

Independent Variables	Symbol	Variable Levels		
		−1	0	1
Temperature (°C)	*X*_1_	60	65	70
Mass ratio of PLand CH	*X*_2_	3:1	4:1	5:1
HT mass (mg)	*X*_3_	2	4	6

## Abbreviations

HT	hydroxytyrosol
RSM	response surface methodology
PL	phospholipid
CH	cholesterol
EE	encapsulation efficiency
DPPH	1,1-diphenyl-2-picrylhydrazyl
ANOVA	analysis of variance
W/O	water-in-oil
MWCO	molecular weight cut off
DLS	dynamic light scattering
